# The Effect of an Essential Oil Blend on Growth Performance, Intestinal Health, and Microbiota in Early-Weaned Piglets

**DOI:** 10.3390/nu15020450

**Published:** 2023-01-14

**Authors:** Yirui Shao, Qingyun Peng, Yuliang Wu, Changfeng Peng, Shanshan Wang, Lijun Zou, Ming Qi, Can Peng, Hongnan Liu, Rui Li, Xia Xiong, Yulong Yin

**Affiliations:** 1 CAS Key Laboratory of Agro-Ecological Processes in Subtropical Region, Hunan Province Key Laboratory of Animal Nutritional Physiology and Metabolic Process, National Engineering Laboratory for Pollution Control and Waste Utilization in Livestock and Poultry Production, Institute of Subtropical Agriculture, Chinese Academy of Sciences, Changsha 410125, China; 2 University of Chinese Academy of Sciences, Beijing 100008, China; 3 Kemin (China) Technologies Co., Ltd., Zhuhai 519040, China; 4 Laboratory of Animal Nutrition and Human Health, College of Life Sciences, Hunan Normal University, Changsha 410081, China; 5 College of Animal Science and Technology, Hunan Agricultural University, Changsha 410000, China; 6 Institute of Animal Nutrition, Northeast Agricultural University, Harbin 150030, China; 7 Laboratory of Basic Biology, Hunan First Normal University, Changsha 410205, China

**Keywords:** carvacrol, thymol, cinnamaldehyde, weaning stress, intestinal epithelial development, intestinal immunity

## Abstract

Essential oils (EO) are promising feed additives for their antibacterial, antioxidant, and immune-enhancing abilities with low toxicity. Carvacrol, thymol, and cinnamaldehyde are commonly used to synthesize EO. However, few studies focus on combining these three EO in early-weaned piglets. In the present study, 24 piglets weaned at 21 d of age were randomly divided into 2 groups (6 replicate pens per group, 2 piglets per pen). The piglets were fed a basal diet (the control group) and a basal diet supplemented with 400 mg/kg EO (a blend consisting of carvacrol, thymol, and cinnamaldehyde, the EO group) for 28 days. At the end of the experiment, one piglet per pen was randomly chosen to be sacrificed. Growth performance, hematology, plasma biochemical indices, antioxidant capacity, intestinal epithelial development and immunity, colonic volatile fatty acids (VFA), and microbiota were determined. The results indicated that the diet supplemented with EO significantly improved average daily feed intake (ADFI, *p* < 0.01) and average daily gain (ADG, *p* < 0.05) in the day 0 to 28 period. EO supplementation led to a significant decrease in plasma lysozyme (*p* < 0.05) and cortisol levels (*p* < 0.01). Additionally, EO significantly promoted jejunal goblet cells in the villus, jejunal mucosa *ZO-1* mRNA expression, ileal villus height, and ileal villus height/crypt depth ratio in piglets (*p* < 0.05). The ileal mucosal TLR4 and NFκB p-p65/p65 protein expression were significantly inhibited in the EO group (*p* < 0.05). Colonic digesta microbiota analysis revealed that bacteria involving the Erysipelotrichaceae family, *Holdemanella* genus, *Phascolarctobacterium* genus, and *Vibrio* genus were enriched in the EO group. In conclusion, these findings indicate that the EO blend improves ADG and ADFI in the day 0 to 28 period, as well as intestinal epithelial development and intestinal immunity in early-weaned piglets, which provides a theoretical basis for the combined use of EO in weaned piglets.

## 1. Introduction

Early weaning is a universal technique to improve the economic benefit of pig farms. However, early weaning is also one of the most stressful events throughout a pig’s life [1]. Separation of piglets from sows, abrupt transition in diet, increased exposure to pathogens, transportation, new environment, fighting, and social hierarchy stress all contribute to weaning stress [1,2,3,4,5]. Weaning stress leads to structural and functional changes in the intestine, including shorter villus, deeper crypt, impaired barrier function, damaged absorptive capacity, and decreased digestive enzyme activities [6,7]. Further, weaning stress potentiates an impaired antioxidant system and increased inflammation, leading to diarrhea and reduced growth [8,9,10]. Nutritional intervention is an effective and convenient method to alleviate weaning stress. However, effective feed additives, including antibiotics and zinc oxide, are confined in their use, considering bacterial resistance and environmental pollution. Thus, new feed additives that are safer and more environmentally friendly are needed.

Essential oils (EO) are aromatic, volatile oil liquids extracted from plants [11]. EOs possess antibacterial, antioxidant, and immune-enhancing abilities with low toxicity, residue, and pollution [12]. Thus, EOs are promising feed additives to alleviate piglet weaning stress as a substitute for antibiotics. Carvacrol, thymol, and cinnamaldehyde are wildly used EO constituents in animal husbandry. Carvacrol and thymol are phenolic monoterpenoids and cinnamaldehyde is a type of phenylpropene found in plant EOs [13,14,15]. The primary function of these EOs is antibacterial activity via interfering with bacterial cytoplasmic membranes and energy metabolism [15]. Additionally, carvacrol, thymol, and cinnamaldehyde are reported to possess antioxidant, antifungal, anticancer, anti-inflammatory, and antidiabetic capacities [16,17,18,19]. The combined use of these EOs has been explored to exert their potential. Reportedly, the combination of cinnamaldehyde and thymol could improve growth performance, digestibility of nutrients, immunity, total antioxidant capacity, and the development of jejunal mucosa in weaned piglets [20,21,22]. The combination of thymol and carvacrol has contributed to increased growth performance, digestibility, and duodenal villus height in weaned piglets [23]. As for the combination of carvacrol, thymol, and cinnamaldehyde, a previous study revealed that mixed EOs led to increased oxidative stress, nitrogen utilization, and decreased odor emission in weaning piglets [24]. It seems contradictory that the combination of the three EOs harms the piglets. However, the effect of EOs depends largely on the species and concentration. The effect of the combination of these three EOs on early-weaned piglets needs further studies.

Therefore, the present study investigated the effect of the EO blend (a combination of carvacrol, thymol, and cinnamaldehyde) on growth performance, immunity, intestinal epithelial development, and intestinal flora in early-weaned piglets.

## 2. Material and Methods

### 2.1. Experimental Design

A total of 24 piglets (Duroc × Landrace × Yorkshire, 7.57 ± 0.25 kg) weaned at 21 d of age were randomly assigned to 2 groups (6 replicate pens per group, 2 piglets per pen). They were fed either a basal diet (control group) or a basal diet supplemented with 400 mg/kg EO (EO group). Several EO concentrations were chosen in the pre-experiment (Supplementary Table S1) based on the previous study [24], and the 400 mg/kg group was selected for further analysis for its great effect on average daily gain (ADG). The composition and nutrient levels of the basal diet are shown in Table 1 and met the nutrition requirements recommended by the National Research Council [25]. EO used in the present study were provided by Kemin (China) Technologies Co., Ltd., Zhuhai, China (Cinsential^TM^ Dry, the product contains 2.64% carvacrol, 1.34% thymol, and 13.80% cinnamaldehyde). For adaptation, a basal diet was provided to all piglets for 3 d before the experiment. Piglets were housed in pens with plastic slotted floors, feeders, and nipple drinkers. All piglets had free access to feed and water. The experiment was divided into two stages: 1 d to 10 d and 11 d to 28 d.

### 2.2. Sample Collection

At the end of the experiment, one piglet per pen was randomly chosen for sampling. After overnight fasting, piglets were euthanized with an intravenous injection of 4% sodium pentobarbital solution (40 mg/kg body weight; Sigma, St. Louis, MO, USA) [26]. Before sacrifice, the blood sample (EDTA and heparin) was collected from the anterior vena cava of piglets. The heparin samples were centrifuged at 3000× *g* at 4 °C for 10 min to obtain plasma and were then frozen in aliquots. The heart, liver, spleen, and kidney were obtained and weighed. Segments of the jejunum and ileum were fixed in 4% paraformaldehyde. Further, jejunal and ileal mucosa cell layers were scraped off, snap-frozen in liquid nitrogen, and stored at −80 °C. Colonic digesta was collected, frozen, and stored at −80 °C.

### 2.3. Growth Performance, Fecal Score, and Organ Index

Body weight was measured every 2 weeks, and feed intake was recorded every day for the calculation of ADG, average daily feed intake (ADFI), and feed efficiency (feed/gain, F/G). Diarrhea was evaluated daily by observers blinded to the treatment using fecal scores (0, normal, firm feces; 1, possible slight diarrhea; 2, definitely unformed, moderately fluid feces; 3, very watery and frothy diarrhea) for each pen [27]. Organ indexes were calculated as the percentage of body weight.

### 2.4. Determination of Hematology and Plasma Biochemical Parameters

The EDTA blood samples were analyzed with a Sysmex KX-21 Hematology Analyzer (Kobe, Japan) to determine white blood cell count (WBC), neutrophil (Neu), lymphocyte (Lym), monocyte (Mon), eosinophil (Eos), and basophil (Bas) content. Plasma cholesterol, triglyceride (TG), high-density lipoprotein cholesterol (HDL-C), and low-density lipoprotein cholesterol (LDL-C) were determined by Cobas C311 Analyser (Roche Diagnostics, Basel, Switzerland). Plasma immunoglobulin A (IgA; Cusabio Biotech Co., Wuhan, China), immunoglobulin M (IgM; Cusabio Biotech Co., Wuhan, China), immunoglobulin G (IgG; Cusabio Biotech Co., Wuhan, China), cortisol (Cusabio Biotech Co., Wuhan, China), and lysozyme (Wuhan Fine Biotech Co., Ltd., Wuhan, China) were determined using commercial ELISA kits.

Plasma total antioxidant capacity (T-AOC; Nanjing Jiancheng Bioengineering Institute, Nanjing, China), total superoxide dismutase (SOD; Beyotime, China), glutathione peroxidase (GSH-Px; Nanjing Jiancheng Bioengineering Institute, Nanjing, China), and malondialdehyde (MDA; Beyotime, China) were assessed using commercial kits.

### 2.5. Phenotype of the T-Lymphocytes

Whole EDTA blood was used for T-lymphocytes phenotype analysis following the procedures based on a previous study [28]. Subpopulations were specified using antibodies against CD3, CD4, and CD8 (Pig CD3e PE, Pig CD8a Alexa 647, and Pig CD4a PerCP-Cy5.5 were all from BD Biosciences, New York, NY, USA). After erythrocytes were lysed using erythrocyte lysate (CwBio, Beijing, China), samples were centrifuged at 450× *g* at 4 °C for 10 min and suspended with PBS to obtain leukocytes. Leukocytes were labeled using antibodies for 30 min on the ice. Single-labeled and non-labeled samples were used as controls. At least 10,000 cells were detected per sample using a Beckman MoFlo XDP flow cytometer (Beckman, Germany).

### 2.6. Intestinal Morphology

Hematoxylin-eosin staining was applied to observe jejunal and ileal morphology. Alcian blue and periodic acid-Schiff staining was applied to count goblet cells [29]. Fixed samples were dehydrated, paraffin-embedded, sectioned, and stained as described in a previous study [30]. Images were acquired with 100 × magnification using an Olympus BX51 microscope (Olympus, Japan). Villus height, crypt depth, and goblet cell numbers were evaluated using the Image-Pro Plus 6.0 image processing and analysis system [31]. Five randomly selected fields were selected for villus height and crypt depth analysis. Goblet cells were counted manually in 10 complete villi or crypts.

### 2.7. Real-Time Quantitative Polymerase Chain Reaction (RT-qPCR)

RNA extraction and RT-qPCR were conducted according to a previous study [32]. Briefly, total RNA was isolated from jejunal and ileal mucosa using AG RNAex Pro Reagent (Accurate Biotechnology [Hunan] Co., Ltd., Changsha, China). The concentration and quality of RNA were assessed using NanoDrop ND-2000 Spectrophotometer (Thermo Fisher Scientific, Waltham, MA, USA) and the 1% agarose electrophoresis. First-strand synthesis of complementary DNA was performed using the Evo M-MLV RT Kit with gDNA Clean for qPCR (Accurate Biotechnology [Hunan] Co., Ltd.). Primers used in RT-qPCR were designed using the NCBI online primer design tool (Primer-BLAST: http://www.ncbi.nlm.nih.gov/tools/primer-blast/ (accessed on 1 July 2021)) according to the gene sequence of the pig or selected from published references (Supplementary Table S2). The RT-qPCR was performed using SYBR Green Premix Pro Taq HS qPCR Kit (Accurate Biotechnology [Hunan] Co., Ltd.) on a LightCycler480 Real-Time PCR system (Roche Diagnostics, Germany). The relative gene expression was normalized by GAPDH using the 2^−ΔΔCt^ method [33]. Data were displayed as the relative values to the control group.

### 2.8. Western Blotting Analysis

The ileal mucosa samples were homogenized with RIPA buffer and the total protein concentration was determined using the BCA method following the manufacturer’s instructions (Beyotime Institute of Biotechnology). The protein expression was detected following the procedures described previously [34]. The antibodies used in the present study were as follows: nuclear factor kappa B (NF-κB) p65 (1:1000, Cell Signaling Technology, MA, USA), phosphor (p)-NF-κB p65 (1:1000, Cell Signaling Technology, MA, USA), IκB kinase (IKK [1:1000, Abcam, Cambridge, UK]), phosphor (p)-IKK (1:1000, Cell Signaling Technology, MA, USA), toll-like receptor 4 (TLR4 [1:500; Proteintech, IL, USA]), β-actin (1:5000; Proteintech, IL, USA), and secondary antibody Goat anti-Mouse or Rabbit IgG (H+L) Secondary Antibody (1:5000, Abiowell, Hunan, China). The protein content was normalized to β-actin, and data were displayed as the relative values to the control group.

### 2.9. Determination of Volatile Fatty Acids (VFA)

VFA concentration in colonic digesta was determined. Briefly, frozen digesta were defrosted, and approximately a 1.00 g sample was taken and weighed. Samples were mixed thoroughly with ddH_2_O and centrifuged at 13,751× *g* for 10 min to obtain the supernatant. The supernatant was mixed with 25% metaphosphoric acid solution (9:1, vol/vol). The mixture was incubated at room temperature for 4 h and filtered with a 0.45μm polysulfide membrane. The VFA concentration was determined using an Agilent 7890A gas chromatograph coupled with an Agilent 5975C mass spectrometer (Agilent Technologies, Santa Clara, CA, USA).

### 2.10. Colonic Digesta Microbiota Analysis

Microbiota analysis was conducted as described in our previous study [35]. Bacterial DNA from colonic content was extracted and amplified using specific primers with barcodes (16S V3 + V4). Illumina NovaSeq PE250 platform (Illumina, San Diego, CA, USA) was used to conduct paired-end sequencing. Raw tags were assembled and filtered to obtain clean data using fqtrim (version 0.9.4) and Vsearch (version 2.3.4). Sequences were assigned to the same operational taxonomic units (OUT) at a 97% similarity level using the UPARSE (version 7.0.1001) [36]. Alpha and beta community diversity was determined using the QIIME2.

### 2.11. Statistical Analysis

Statistical analysis and diagram visualization were completed using GraphPad Prism (version 8.0.2) and R software (version 3.5.2). The student’s unpaired *T*-test was used to assess differences between the two groups after the normality of data was evaluated with the Shapiro–Wilk *W*-test and the potential outliers were evaluated with ROUT analysis. Data were expressed as means ± standard error of the mean (SEM), 0.05 < *p* < 0.1 indicated a trend toward significance, 0.01 ≤ *p* < 0.05 was considered significant, and *p* < 0.01 was considered extremely significant.

## 3. Results

### 3.1. Effect of EO on Piglet Growth Performance

The effects of dietary supplementation of EO on the growth performance of weaned piglets are shown in Table 2. Compared to the control group, the ADG from 0 to 28 d (*p* < 0.05) and the ADFI during different periods (*p* < 0.05) were significantly higher in the EO group. Additionally, the piglets fed the EO diet had a higher body weight on day 28 (*p* < 0.1). However, the body weight and F/G were comparable between the two groups (*p* > 0.05).

### 3.2. Effect of EO on Piglet Diarrhea Score and Organ Index

**Figure 1:**
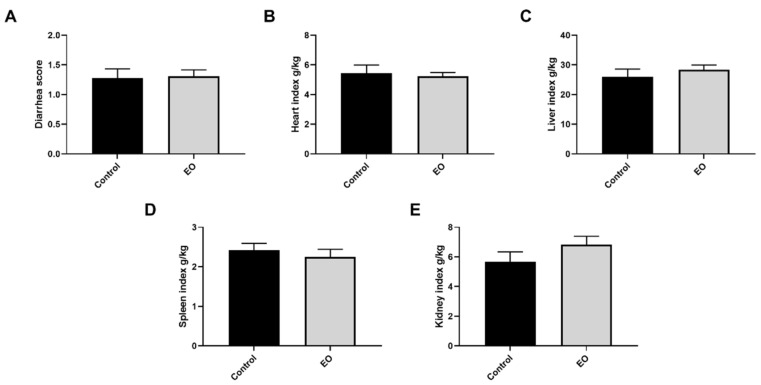
Effect of EO on piglet diarrhea score (**A**) and organ index (**B**–**E**). Data are presented as means ± SEM (*n* = 6). EO, essential oil.

The diarrhea score for the entire experiment period was calculated. There was no significant difference in diarrhea score and organ index between the two groups (*p* > 0.05, Figure 1).

### 3.3. Effect of EO on Piglet Hematology, Plasma Biochemical Index, and Antioxidant Capacity

**Figure 2:**
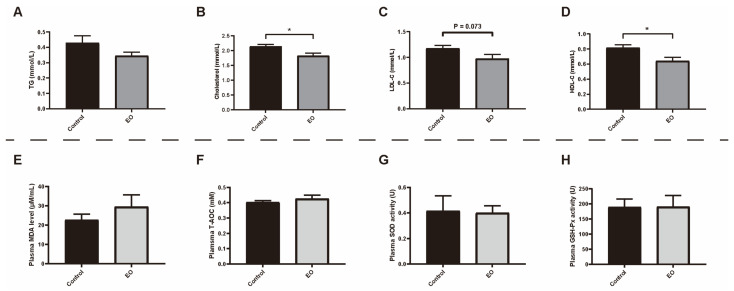
Effect of EO on piglet plasma biochemical index (**A**–**D**) and antioxidant capacity (**E**–**H**). Data are presented as means ± SEM (*n* = 6). * *p* < 0.05. EO: essential oil; TG: triglycerides; LDL-C: low density lipoprotein cholesterol; HDL-C: high density lipoprotein cholesterol; EO: essential oil; MDA: malondialdehyde; T-AOC: total antioxidant capacity; SOD: superoxide dismutase; GSH-Px: glutathione peroxidase.

There was no significant difference in the hematology index between the two groups (*p* > 0.05, Supplementary Table S3). EO supplementation significantly decreased the plasma cholesterol and HDL-C level (*p* < 0.05) compared to the control group (Figure 2B,D). The plasma LDL-C in the EO group was lower than in the control group (*p* < 0.1, Figure 2C). Additionally, there was no significant difference in plasma MDA level, SOD activity, GSH-Px activity, and T-AOC between the two groups (*p* > 0.05; Figure 2E–H).

### 3.4. Effect of EO on Piglet Immunity

**Figure 3:**
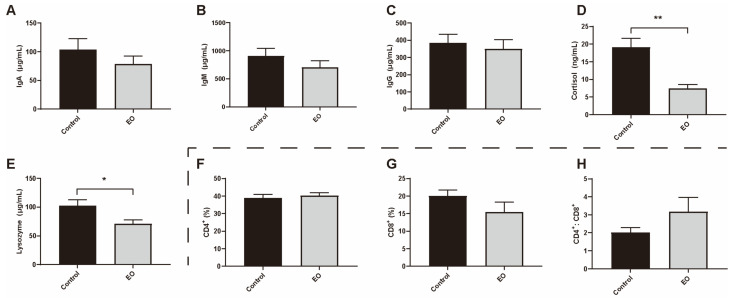
Effect of EO on piglet plasma immunity parameters. (**A**–**C**) Effect of EO on piglet plasma immunoglobulin. (**D**) Effect of EO on piglet plasma cortisol level. (**E**) Effect of EO on piglet plasma lysozyme level. (**F**–**H**) Effect of EO on piglet blood lymphocyte subset. Data are presented as means ± SEM (*n* = 6). * *p* < 0.05, ** *p* < 0.01. EO: essential oil; IgA: immunoglobulin A; IgM: immunoglobulin M; IgG: immunoglobulin G.

Compared to the control group, the plasma cortisol (*p* < 0.01) and lysozyme (*p* < 0.05) levels were significantly decreased in the EO group (Figure 3D,E). There was no significant difference in blood lymphocyte subsets between these two groups (Figure 3F–H).

### 3.5. Effect of EO on Piglet Intestinal Epithelial Morphological Structure

**Figure 4:**
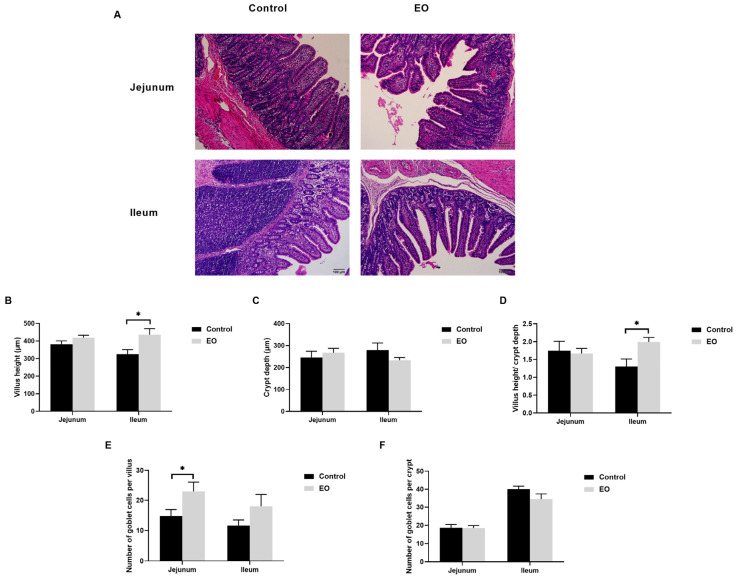
Effect of EO on piglet jejunal and ileal epithelial morphological structure. (**A**) Representative hematoxylin–eosin-stained image of piglet jejunum and ileum (scale bar: 100 μm). (**B**–**D**) Villus height, crypt depth, and villus height/crypt depth ratio of piglet jejunum and ileum. (**E**) The number of goblet cells per villus in the jejunum and ileum. (**F**) The number of goblet cells per crypt in jejunum and ileum. Data are presented as means ± SEM (*n* = 6). * *p* < 0.05. EO: essential oil.

The ileal villus height and villus height/crypt depth ratio in the EO group were significantly higher than the control group (*p* < 0.05; Figure 4A–D). Moreover, there was no significant difference in piglet jejunum (*p* > 0.05; Figure 4A–D). The number of goblet cells in the jejunal villus was significantly higher in the EO group than in the control group (*p* < 0.05; Figure 4E). Furthermore, there was no significant difference in piglet ileum (*p* > 0.05; Figure 4E,F).

### 3.6. Effect of EO on Piglet Intestinal Barrier Function

**Figure 5:**
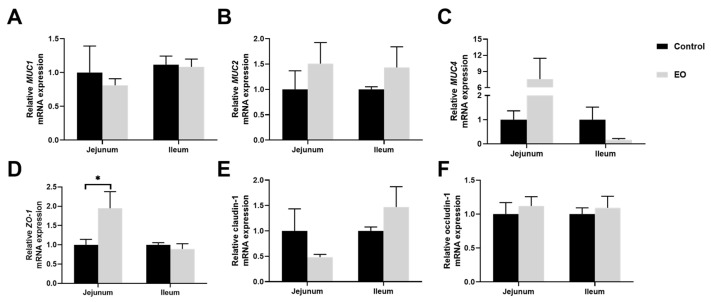
Effect of EO on relative mRNA expression of genes involved in barrier function in piglet jejunal and ileal mucosa (**A**–**F**). Data are presented as means ± SEM (*n* = 6), * *p* < 0.05. EO: essential oil. *MUC1*: mucin-1; *MUC2*: mucin-2; *MUC4*: mucin-4; *ZO-1*: zonula occludens-1.

The relative mRNA expression of *ZO-1* was significantly higher in jejunal mucosa in the EO group than in the control group (*p* < 0.05; Figure 5D). And there was no significant difference in *MUC1*, *MUC2*, *MUC4*, claudin-1, or occludin-1 mRNA expression (*p* > 0.05, Figure 5).

### 3.7. Effect of EO on Piglet Ileum Immune Function

**Figure 6:**
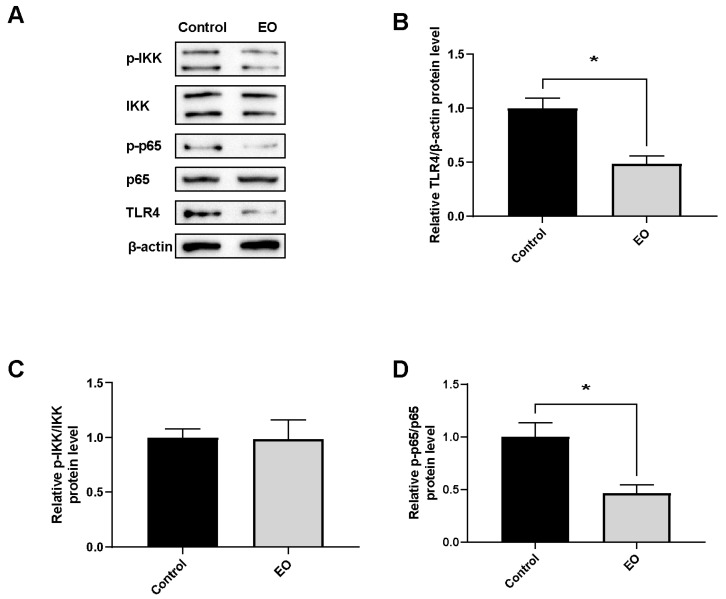
Effect of EO on relative protein expression of genes involved in NF-κB pathway in ileal mucosa (**A**–**D**). Data are presented as means ± SEM (*n* = 4), * *p* < 0.05. EO: essential oil; p-IKK: phosphor-IκB kinase; IKK: IκB kinase; p-p65: phosphor-nuclear factor kappa B; p65: nuclear factor kappa B; TLR4: toll-like receptor 4.

Relative protein expression of genes involved in the NF-κB pathway was determined in piglet ileal mucosa (Figure 6). EO supplement significantly decreased TLR4 and NFκB p-p65/p65 protein expression (*p* < 0.05).

### 3.8. Effect of EO on VFA Concentrations and Microbiome in Piglet Colonic Content

**Figure 7:**
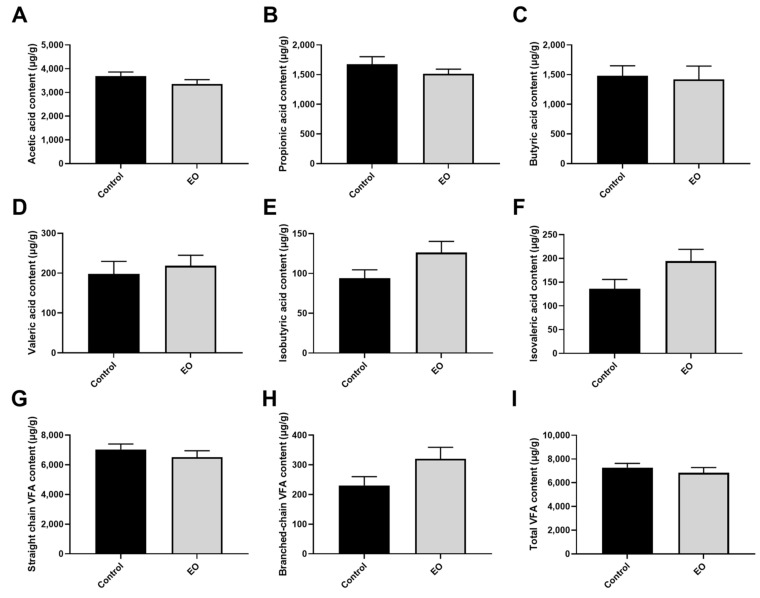
Effect of EO on VFA concentrations in piglet colonic content (**A**–**I**). Data are presented as means ± SEM (*n* = 6). EO: essential oil; VFA: volatile fatty acid.

**Figure 8:**
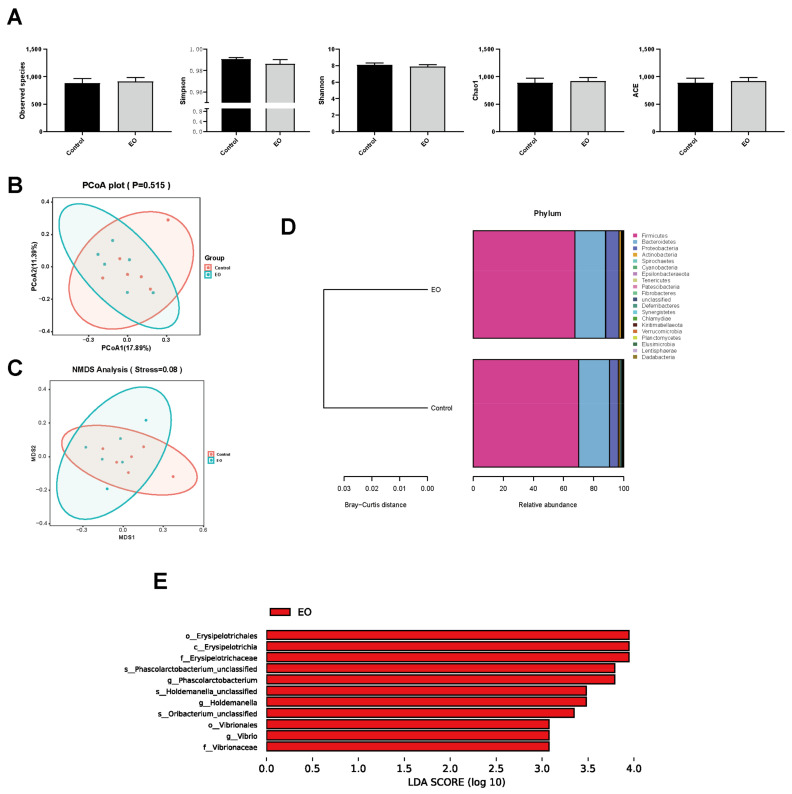
Effect of EO on microbial α-diversity and β-diversity in piglet colonic content. (**A**) Effect of EO on microbial α-diversity. (**B**,**C**) PCoA and NMDS analyses. (**D**) UPGMA analysis. (**E**) LEfSe analysis. Data are presented as means ± SEM (*n* = 6). EO: essential oil; PCoA: principal coordinate analysis; NMDS: non-metric multidimensional scaling; UPGMA: unweighted pair-group method with arithmetic mean; LEfSe: linear discriminant analysis effect size.

In total, 12 colonic content samples were used for VFA concentration determination and 16S rDNA sequencing. The difference between the two groups in VFA concentrations in piglet colonic content was insignificant (*p* > 0.05; Figure 7). Microbial α-diversity and β-diversity analysis revealed few differences between the two groups (Figure 8A–C). The unweighted pair-group method with arithmetic mean (UPGMA) analysis disclosed similar microbial composition between the two groups (Figure 8D). The core floras were composed of Firmicutes, Bacteroidetes, and Proteobacteria. LEfSe analysis revealed that the Erysipelotrichaceae family, *Phascolarctobacterium*, *Holdemanella*, and *Vibrio* genus were the biomarker bacteria in the EO group (Figure 8E).

## 4. Discussion

Weaning stress contributes to inflammation and oxidative stress, leading to an impaired intestinal barrier [10,37]. EOs are effective additives to alleviate weaning stress in early-weaned piglets without resistance and environmental pollution [12]. The use of a single EO has drawn the attention of researchers; however, the combined use of different EOs needs further exploration. In the present study, we assessed the effect of an EO blend on growth performance, plasma biochemical index, immunity, intestinal epithelial morphology, barrier function, and microbiota in early-weaned piglets.

Early weaning leads to descending feed intake and decreased growth rate, owing to poor palatability and digestibility of the solid dry diet [38]. In this study, EO supplementation significantly improved ADG and ADFI with few changes in F/G. It agreed with the previous study that combining thymol and cinnamaldehyde led to a significant increase in ADG and ADFI in weaning piglets [39]. A possible explanation for the increased ADFI might be related to the pleasant odor and flavor of EOs, which contributed to improved appetite [40].

The separation from sows and the abrupt change in diet might be a stimulus for declined villus height and increased crypt depth in weaning piglets [41,42]. Declined villus height is mainly induced by aggravated apoptosis and compromised renewal of intestinal epithelial cells under stresses or diseases [43]. In this study, EO supplementation led to increased villus height and villus height/crypt depth ratio. In line with this, carvacrol and thymol diets enhanced the villus height/crypt depth ratio in the distal small intestine of weaned piglets [44]. However, the EO blend (a combination of thymol and cinnamaldehyde) significantly increased the villus height/crypt depth ratio in the weaned piglet jejunum instead of the ileum [22]. Different EO types and concentrations might cause different effects. Goblet cells synthesize and secret complex mucins to protect the intestinal epithelium from pathogens and toxins [45]. A tight junction is indispensable for the integrity of intestinal epithelial barrier, including occludin, zonula occludens (ZO), and claudins [46]. In this study, EO supplementation significantly augmented jejunal goblet cells in the villus, and jejunal *ZO-1* mRNA expression, which agreed with the previous study that carvacrol administration significantly increased *ZO-1* mRNA expression and goblet cells in the broiler small intestine [47]. Collectively, these data suggested that the EO diet contributed to ileal epithelial development and jejunal epithelial integrity.

Carvacrol and thymol possess an inhibitory effect on 3-hydroxy-3-methylglutaryl coenzyme A reductase, the rate-limiting enzyme of cholesterol synthesis [48]. Reducing plasma cholesterol and HDL-C levels might be related to the hypocholesterolemic effect of carvacrol and thymol. Elevated cortisol is a stress biomarker, and the cortisol level is increased in weaned piglets suffering from weaning stress [49]. Lysozyme is secreted by monocyte–macrophage and epithelioid cells [50]. Reportedly, an augmented serum lysozyme level was found in patients with Crohn’s disease and ulcerative colitis [51]. Our results showed that EO administration decreased plasma cortisol and lysozyme levels, suggesting EO relieved weaning stress in piglets. The intestine is the principal organ involved in immunity, in which the ileum plays an indispensable role. Reportedly, weaning stress has led to a severe inflammatory immune response in the piglet intestine [52], contributing to disturbed intestinal function and retarded growth performance [53,54]. The anti-inflammatory effect has been found in various EO. Cinnamic aldehyde exerted anti-inflammatory effects by targeting TLR2, TLR4, and NFκB; oregano extracts (containing sabinene hydrate, thymol, and carvacrol) relieve inflammation by decreasing pro-inflammatory cytokines and increasing anti-inflammatory cytokine synthesis [55]; cinnamaldehyde relieves inflammation by suppressing NO release, decreasing COX-2 expression, and increasing cAMP production [56]. In this study, EO supplementation inhibited TLR4 and NFκB p-p65/p65 protein expression in ileal mucosa. Collectively, EO supplementation relieved piglet stress and suppressed the ileal inflammatory TLR4/NFκB pathway.

The intestinal microbiota closely interacts with intestinal immunity [57]. In this study, little change was observed in colon microbial α-diversity and β-diversity, consistent with the previous study [24]. Bacteroidetes, Firmicutes, Proteobacteria, and Actinobacteria are the predominant phyla in most mammals [58], which agreed with our study that these phyla were the most abundant in piglet colonic digesta. The relative abundance of Erysipelotrichaceae and *Phascolarctobacterium* is negatively related to inflammatory disease. Reportedly, the relative abundance of Erysipelotrichaceae in piglet cecum contents of digesta was negatively correlated with the expression of inflammatory factors [59]. *Phascolarctobacterium* was positively correlated with serum antioxidant capacity, and negatively correlated with serum pro-inflammatory cytokines in piglets [60]. However, the effect of *Holdemanella* is controversial. A previous study reported that the abundance of *Holdemanella* in piglet cecum content was positively related to serum pro-inflammatory cytokines [61], whereas others reported that *Holdemanella* showed anti-inflammatory activity in colitis patients [62]. *Vibrio* is a common pathogen that contributes to diseases such as intestinal inflammation and diarrhea [63]. Our result showed that Erysipelotrichaceae and *Phascolarctobacterium* were significantly enriched in the EO group, which might be related to the down-regulated inflammatory pathway and mitigated stress in piglets. Few studies focus on the exact function of EOs on these bacteria, and the underlying mechanism needs further exploration. Collectively, the microbiota changes induced by EO supplementation might be related to inhibited inflammatory pathways in piglets.

In conclusion, this study reported changes in growth performance, immunity, intestinal epithelial development, and intestinal flora in early-weaned piglets, caused by EO supplementation. Our results demonstrated that EO supplementation contributed to improved ADG and ADFI in the day 0 to 28 period, less stress, improved ileal epithelial development, and suppression of the ileal inflammatory TLR4/NFκB pathway. Additionally, EOs led to few changes in microbiota composition, and the enriched flora in the EO group might be related to depressed inflammatory pathways in piglets. This study provides a theoretical basis for the combined use of EO. However, further studies are needed to explain the underlying mechanism.

## Figures and Tables

**Table 1:** Composition and nutrient levels of experimental diets.

Composition, %	Phase 1, Days 1–10	Phase 2, Days 11–28
Extruded corn	30.53	30
Corn	25	32.42
Soybean meal	8.55	10.1
Soybean protein concentrate	8	8
Whey powder	8	5
Peru fish meal	6	5
Limestone	0.2	0.2
Soybean oil	2	2
Sucrose	2	-
Glucose	5	3
Calcium formate	0.6	0.6
Calcium hydrogen phosphate	0.5	0.4
Choline chloride	0.1	0.1
Premix ^1^	1	1
Antioxidant	0.05	0.05
Citric acid	0.8	0.8
ZnO	0.2	0.02
Salt	0.4	0.4
Lysine	0.62	0.55
DL-methionine	0.09	0.07
Threonine	0.25	0.2
Tryptophan	0.06	0.04
Fungicide	0.05	0.05
Total	100	100
Calculated levels, %		
CP	19	18.5
Ca	0.79	0.7
AP	0.44	0.38
Lysine	1.35	1.23
Methionine	0.39	0.36
Threonine	0.79	0.73
Tryptophan	0.22	0.2
DE MJ/kg	14.67	14.57

^1^ The premix provided the following vitamins and minerals per kilogram of the diet: vitamin A 6450 IU, vitamin D_3_ 2250 IU, vitamin E 25 IU, vitamin K 3 mg, vitamin B_1_ 1.8 mg, vitamin B_12_ 0.026 mg, riboflavin 8 mg, folic acid 0.9 mg, biotin 4.5 mg, niacin 24 mg, pantothenic acid 20 mg, Zn 80 mg, Fe 150 mg, Cu 10 mg, Mn 4 mg, I 0.6 mg, Se 0.5 mg, Co 0.8 mg. CP: crude protein; Ca: calcium; AP: available phosphorus; DE: digestible energy.

**Table 2:** Effect of EO on growth performance in early-weaned piglets.

	Control	EO	*p*-Value
Body weight (kg)			
Day 0	7.37 ± 0.21	7.77 ± 0.45	0.436
Day 14	9.74 ± 0.39	10.43 ± 0.62	0.364
Day 28	15.30 ± 0.62	17.82 ± 1.12	0.077
ADG (g/d)			
Day 0 to 14	169.35 ± 16.16	190.18 ± 14.86	0.365
Day 14 to 28	430.00 ± 11.59	527.38 ± 39.88	0.082
Day 0 to 28	283.33 ± 16.49	358.78 ± 26.49	0.036
ADFI (g/d)			
Day 0 to 14	373.39 ± 16.39	488.54 ± 26.72	0.004
Day 14 to 28	892.56 ± 38.65·	1092.76 ± 51.73	0.011
Day 0 to 28	632.98 ± 25.11	790.65 ± 37.94	0.006
F/G			
Day 0 to 14	2.33 ± 0.27	2.63 ± 0.21	0.399
Day 14 to 28	2.13 ± 0.04	2.10 ± 0.10	0.807
Day 0 to 28	2.25 ± 0.08	2.24 ± 0.12	0.921

Data are presented as means ± SEM (*n* = 6). EO: essential oil; ADG: average daily gain; ADFI: average daily feed intake; F/G: feed/gain ratio.

## Data Availability

The assembled HiSeq sequences obtained in the present study were submitted to NCBI Sequence Read Archive (SRA, No. PRJNA898675).

## Supplementary Materials

The following supporting information can be downloaded at: https://www.mdpi.com/article/10.3390/nu15020450/s1, Table S1: Effect of different concentrations of EO on growth performance in early-weaned piglets.; Table S2: Primer pairs used for the real-time quantitative polymerase chain reaction; Table S3. Effect of EO on piglet hematology index. References [64,65,66,67,68] are cited in the supplementary materials.

## References

[1] Campbell J.M., Crenshaw J.D., Polo J. (2013). The biological stress of early weaned piglets. J. Anim. Sci. Biotechnol..

[2] Lallès J.P., Boudry G., Favier C., Le Floc’h N., Luron I., Montagne L., Oswald I.P., Pié S., Piel C., Sève B. (2004). Gut function and dysfunction in young pigs: Physiology. Anim. Res..

[3] Lewis N.J. (2008). Transport of early weaned piglets. Appl. Anim. Behav. Sci..

[4] Parratt C.A., Chapman K.J., Turner C., Jones P.H., Mendl M.T., Miller B.G. (2006). The fighting behaviour of piglets mixed before and after weaning in the presence or absence of a sow. Appl. Anim. Behav. Sci..

[5] Sutherland M.A., Backus B.L., McGlone J.J. (2014). Effects of transport at weaning on the behavior, physiology and performance of pigs. Animals.

[6] Zhu L., Zhao K., Chen X., Xu J. (2012). Impact of weaning and an antioxidant blend on intestinal barrier function and antioxidant status in pigs. J. Anim. Sci..

[7] Wijtten P.J., van der Meulen J., Verstegen M.W. (2011). Intestinal barrier function and absorption in pigs after weaning: A review. Br. J. Nutr..

[8] Yin J., Wu M.M., Xiao H., Ren W.K., Duan J.L., Yang G., Li T.J., Yin Y.L. (2014). Development of an antioxidant system after early weaning in piglets2. J. Anim. Sci..

[9] Pohl C.S., Medland J.E., Mackey E., Edwards L.L., Bagley K.D., DeWilde M.P., Williams K.J., Moeser A.J. (2017). Early weaning stress induces chronic functional diarrhea, intestinal barrier defects, and increased mast cell activity in a porcine model of early life adversity. Neurogastroenterol. Motil..

[10] Novais A.K., Deschene K., Martel-Kennes Y., Roy C., Laforest J.P., Lessard M., Matte J.J., Lapointe J. (2021). Weaning differentially affects mitochondrial function, oxidative stress, inflammation and apoptosis in normal and low birth weight piglets. PLoS ONE.

[11] Brenes A., Roura E. (2010). Essential oils in poultry nutrition: Main effects and modes of action. Anim. Feed Sci. Technol..

[12] Zeng Z., Zhang S., Wang H., Piao X. (2015). Essential oil and aromatic plants as feed additives in non-ruminant nutrition: A review. J. Anim. Sci. Biotechnol..

[13] Tang X., Chen S., Wang L. (2011). Purification and identification of carvacrol from the root of Stellera chamaejasme and research on its insecticidal activity. Nat. Prod. Res..

[14] Mancini E., Senatore F., Del Monte D., De Martino L., Grulova D., Scognamiglio M., Snoussi M., De Feo V. (2015). Studies on chemical composition, antimicrobial and antioxidant activities of five *Thymus vulgaris* L. essential oils. Molecules.

[15] Omonijo F.A., Ni L., Gong J., Wang Q., Lahaye L., Yang C. (2018). Essential oils as alternatives to antibiotics in swine production. Anim. Nutr..

[16] Kowalczyk A., Przychodna M., Sopata S., Bodalska A., Fecka I. (2020). Thymol and Thyme Essential Oil—New Insights into Selected Therapeutic Applications. Molecules.

[17] Salehi B., Mishra A.P., Shukla I., Sharifi-Rad M., Contreras M.d.M., Segura-Carretero A., Fathi H., Nasrabadi N.N., Kobarfard F., Sharifi-Rad J. (2018). Thymol, thyme, and other plant sources: Health and potential uses. Phytother. Res..

[18] Suntres Z.E., Coccimiglio J., Alipour M. (2015). The Bioactivity and Toxicological Actions of Carvacrol. Crit. Rev. Food Sci. Nutr..

[19] Hajinejad M., Ghaddaripouri M., Dabzadeh M., Forouzanfar F., Sahab-Negah S. (2020). Natural Cinnamaldehyde and Its Derivatives Ameliorate Neuroinflammatory Pathways in Neurodegenerative Diseases. BioMed Res. Int..

[20] Li P., Piao X., Ru Y., Han X., Xue L., Zhang H. (2012). Effects of Adding Essential Oil to the Diet of Weaned Pigs on Performance, Nutrient Utilization, Immune Response and Intestinal Health. Asian-Aust. J. Anim. Sci..

[21] Zeng Z., Xu X., Zhang Q., Li P., Zhao P., Li Q., Liu J., Piao X. (2015). Effects of essential oil supplementation of a low-energy diet on performance, intestinal morphology and microflora, immune properties and antioxidant activities in weaned pigs. Anim. Sci. J..

[22] Tian Q., Piao X. (2019). Essential oil blend could decrease diarrhea prevalence by improving antioxidative capability for weaned pigs. Animals.

[23] Xu Y.T., Liu L., Long S.F., Pan L., Piao X.S. (2018). Effect of organic acids and essential oils on performance, intestinal health and digestive enzyme activities of weaned pigs. Anim. Feed Sci. Technol..

[24] Mo K.B., Li J., Liu F.F., Xu Y., Huang X.H., Ni H.J. (2022). Superiority of Microencapsulated Essential Oils Compared with Common Essential Oils and Antibiotics: Effects on the Intestinal Health and Gut Microbiota of Weaning Piglet. Front Nutr..

[25] NRC (2012). Nutrient Requirements of Swine.

[26] Wang Q., Xiong X., Li J., Tu Q., Yang H., Yin Y. (2018). Energy metabolism in the intestinal crypt epithelial cells of piglets during the suckling period. Sci. Rep..

[27] Yu J., Song Y., Yu B., He J., Zheng P., Mao X., Huang Z., Luo Y., Luo J., Yan H. (2020). Tannic acid prevents post-weaning diarrhea by improving intestinal barrier integrity and function in weaned piglets. J. Anim. Sci. Biotechnol..

[28] Li J., Zhong Y., Li H., Zhang N., Ma W., Cheng G., Liu F., Liu F., Xu J. (2011). Enhancement of Astragalus polysaccharide on the immune responses in pigs inoculated with foot-and-mouth disease virus vaccine. Int. J. Biol. Macromol..

[29] Deng Q., Shao Y., Wang Q., Li J., Li Y., Ding X., Huang P., Yin J., Yang H., Yin Y. (2020). Effects and interaction of dietary electrolyte balance and citric acid on the intestinal function of weaned piglets. J. Anim. Sci..

[30] Wang J., Zeng L., Tan B., Li G., Huang B., Xiong X., Li F., Kong X., Liu G., Yin Y. (2016). Developmental changes in intercellular junctions and Kv channels in the intestine of piglets during the suckling and post-weaning periods. J. Anim. Sci. Biotechnol..

[31] Ruan Z., Liu S., Zhou Y., Mi S., Liu G., Wu X., Wu X., Yao K., Assaad H., Deng Z. (2014). Chlorogenic acid decreases intestinal permeability and increases expression of intestinal tight junction proteins in weaned rats challenged with LPS. PLoS ONE.

[32] Qi M., Tan B., Wang J., Li J., Liao S., Yan J., Liu Y., Yin Y. (2019). Small intestinal transcriptome analysis revealed changes of genes involved in nutrition metabolism and immune responses in growth retardation piglets1. J. Anim. Sci..

[33] Livak K.J., Schmittgen T.D. (2001). Analysis of relative gene expression data using real-time quantitative PCR and the 2−ΔΔCT method. Methods.

[34] Xiao H., Wu M.M., Tan B.E., Yin Y.L., Li T.J., Xiao D.F., Li L. (2013). Effects of composite antimicrobial peptides in weanling piglets challenged with deoxynivalenol: II. Intestinal morphology and function1. J. Anim. Sci..

[35] Qi M., Tan B., Wang J., Liao S., Li J., Cui Z., Shao Y., Ji P., Yin Y. (2021). Postnatal growth retardation is associated with deteriorated intestinal mucosal barrier function using a porcine model. J. Cell Physiol..

[36] Edgar R.C. (2013). UPARSE: Highly accurate OTU sequences from microbial amplicon reads. Nat. Methods.

[37] Smith F., Clark J.E., Overman B.L., Tozel C.C., Huang J.H., Rivier J.E.F., Blisklager A.T., Moeser A.J. (2010). Early weaning stress impairs development of mucosal barrier function in the porcine intestine. Am. J. Physiol. Gastroint. Liver Physiol..

[38] Surai P., Fisinin V. (2015). Antioxidant-prooxidant balance in the intestine: Applications in chick placement and pig weaning. J. Vet. Sci. Med..

[39] Li S.Y., Ru Y.J., Liu M., Xu B., Péron A., Shi X.G. (2012). The effect of essential oils on performance, immunity and gut microbial population in weaner pigs. Livest. Sci..

[40] Ogawa K., Honda M., Tanigawa A., Hatase A., Ito A., Higa Y., Morinaga O. (2020). Appetite-enhancing effects of inhaling cinnamon, clove, and fennel essential oils containing phenylpropanoid analogues. J. Nat. Med..

[41] Pluske J.R. (1993). Psychological and Nutritional Stress in Pigs at Weaning: Production Parameters, the Stress Response, and Histology and Biochemistry of the Small Intestine.

[42] Van Beers-Schreurs H.M.G., Nabuurs M.J.A., Vellenga L., Valk HJK-vd Wensing T., Breukink H.J. (1998). Weaning and the Weanling Diet Influence the Villous Height and Crypt Depth in the Small Intestine of Pigs and Alter the Concentrations of Short-Chain Fatty Acids in the Large Intestine and Blood. J. Nutr..

[43] Van der Peet-Schwering C., Jansman A., Smidt H., Yoon I. (2007). Effects of yeast culture on performance, gut integrity, and blood cell composition of weanling pigs. J. Anim. Sci..

[44] Michiels J., Missotten J., Van Hoorick A., Ovyn A., Fremaut D., De Smet S., Dierick N. (2010). Effects of dose and formulation of carvacrol and thymol on bacteria and some functional traits of the gut in piglets after weaning. Arch. Anim. Nutr..

[45] Liu L., Fu C., Yan M., Xie H., Li S., Yu Q., He S., He J. (2016). Resveratrol modulates intestinal morphology and HSP70/90, NF-κB and EGF expression in the jejunal mucosa of black-boned chickens on exposure to circular heat stress. Food Funct..

[46] Zhang C., Zhao X.H., Yang L., Chen X.Y., Jiang R.S., Jin S.H., Geng Z.Y. (2017). Resveratrol alleviates heat stress-induced impairment of intestinal morphology, microflora, and barrier integrity in broilers. Poult. Sci..

[47] Liu S., Song M., Yun W., Lee J., Lee C., Kwak W., Han N., Kim H., Cho J. (2018). Effects of oral administration of different dosages of carvacrol essential oils on intestinal barrier function in broilers. J. Anim. Physiol. Anim. Nutr..

[48] Elson C.E. (1995). Suppression of mevalonate pathway activities by dietary isoprenoids: Protective roles in cancer and cardiovascular disease. J. Nutr..

[49] Moeser A.J., Klok C.V., Ryan K.A., Wooten J.G., Little D., Cook V.L., Blikslager A. (2007). Stress signaling pathways activated by weaning mediate intestinal dysfunction in the pig. Am. J. Physiol. Gastroint. Liver Physiol..

[50] Bergantini L., Bianchi F., Cameli P., Mazzei M.A., Fui A., Sestini P., Rottoli P., Bargagli E. (2019). Prognostic Biomarkers of Sarcoidosis: A Comparative Study of Serum Chitotriosidase, ACE, Lysozyme, and KL-6. Dis. Markers.

[51] Klass H., Neale G. (1978). Serum and faecal lysozyme in inflammatory bowel disease. Gut.

[52] Pié S., Lalles J.P., Blazy F., Laffitte J., Sève B., Oswald I.P. (2004). Weaning is associated with an upregulation of expression of inflammatory cytokines in the intestine of piglets. J. Nutr..

[53] Sève B. (2000). Effects of underfeeding during the weaning period on growth, metabolism, and hormonal adjustments in the piglet. Domest. Anim. Endocrinol..

[54] Boudry G., Péron V., Le Huërou-Luron I., Lalles J.P., Sève B. (2004). Weaning induces both transient and long-lasting modifications of absorptive, secretory, and barrier properties of piglet intestine. J. Nutr..

[55] Ocana-Fuentes A., Arranz-Gutierrez E., Senorans F., Reglero G. (2010). Supercritical fluid extraction of oregano (Origanum vulgare) essentials oils: Anti-inflammatory properties based on cytokine response on THP-1 macrophages. Food Chem. Toxicol..

[56] Ballabeni V., Tognolini M., Giorgio C., Bertoni S., Bruni R., Barocelli E. (2010). Ocotea quixos Lam. essential oil: In vitro and in vivo investigation on its anti-inflammatory properties. Fitoterapia.

[57] Maynard C.L., Elson C.O., Hatton R.D., Weaver C.T. (2012). Reciprocal interactions of the intestinal microbiota and immune system. Nature.

[58] Ley R.E., Hamady M., Lozupone C., Turnbaugh P.J., Ramey R.R., Bircher J.S., Schlegel M.L., Tucker T.A., Schrenzel M.D., Knight R. (2008). Evolution of mammals and their gut microbes. Science.

[59] Zhou J., Luo J., Yang S., Xiao Q., Wang X., Zhou Z., Xiao Y., Shi D. (2021). Different Responses of Microbiota across Intestinal Tract to Enterococcus faecium HDRsEf1 and Their Correlation with Inflammation in Weaned Piglets. Microorganisms.

[60] Jiang Z., Su W., Li W., Wen C., Du S., He H., Zhang Y., Gong T., Wang X., Wang Y. (2022). Bacillus amyloliquefaciens 40 regulates piglet performance, antioxidant capacity, immune status and gut microbiota. Anim. Nutr..

[61] Hu R., He Z., Liu M., Tan J., Zhang H., Hou D.-X., Wu S. (2020). Dietary protocatechuic acid ameliorates inflammation and up-regulates intestinal tight junction proteins by modulating gut microbiota in LPS-challenged piglets. J. Anim. Sci. Biotechnol..

[62] Pujo J., Petitfils C., Le Faouder P., Eeckhaut V., Payros G., Maurel S., Perez-Berezo T., van Hul M., Barreau F., Blanpied C. (2021). Bacteria-derived long chain fatty acid exhibits anti-inflammatory properties in colitis. Gut.

[63] Ritchie J.M., Rui H., Zhou X., Iida T., Kodoma T., Ito S., Davis B.M., Bronson R.T., Waldor M.K. (2012). Inflammation and disintegration of intestinal villi in an experimental model for Vibrio parahaemolyticus-induced diarrhea. PLoS Pathog..

[64] Yin J., Li Y., Han H., Liu Z., Zeng X., Li T., Yin Y. (2018). Long-term effects of lysine concentration on growth performance.; intestinal microbiome.; and metabolic profiles in a pig model. Food Funct..

[65] Pieper R., Kröger S., Richter J.F., Wang J., Martin L., Bindelle J., Htoo J., Von Smolinski D., Vahjen W., Zentek J. (2012). Fermentable fiber ameliorates fermentable protein-induced changes in microbial ecology, but not the mucosal response, in the colon of piglets. J. Nutr..

[66] He L., Zhou X., Huang N., Li H., Cui Z., Tian J., Jiang Q., Liu S., Wu J., Li T. (2017). Administration of alpha-ketoglutarate improves epithelial restitution under stress injury in early-weaning piglets. Oncotarget.

[67] Tao S., Bai Y., Li T., Li N., Wang J. (2019). Original low birth weight deteriorates the hindgut epithelial barrier function in pigs at the growing stage. FASEB J..

[68] Zhou H., Sun J., Ge L., Liu Z., Chen H., Yu B., Chen D. (2020). Exogenous infusion of short-chain fatty acids can improve intestinal functions independently of the gut microbiota. J Anim Sci..

