# Supplementary material for: The Effect of an Essential Oil Blend on Growth Performance, Intestinal Health, and Microbiota in Early-Weaned Piglets
Source: Nutrients. 2023 Jan 14;15(2):450. doi: 10.3390/nu15020450 (PMC9862375; doi:10.3390/nu15020450)
Supplement: Supplementary file 1 [file nutrients-15-00450-s001.zip › nutrients-2139283-supplementary.pdf]

Supplementary Material

Table S1. Effect of different concentration of EO on growth performance in early-weaned piglets.

	Control	0.005% EO	0.01% EO	0.02% EO	0.04% EO	*P*-value
Body weight (kg)						
Day 0	7.37 ± 0.21	7.67 ± 0.42	7.70 ± 0.39	7.70 ± 0.41	7.77 ± 0.45	0.952
Day 14	9.74 ± 0.39	9.87 ± 0.83	10.06 ± 0.63	10.03 ± 0.62	10.43 ± 0.62	0.951
Day 28	15.30 ± 0.62	16.36 ± 1.23	16.58 ± 1.44	16.55 ± 0.89	17.82 ± 1.12	0.623
ADG (g/d)						
Day 0 to 14	169.35 ± 16.16	157.14 ± 32.98	168.45 ± 18.97	166.07 ± 16.1	190.18 ± 14.86	0.850
Day 14 to 28	430.00 ± 11.59	463.69 ± 37.85	465.48 ± 60.77	465.77 ± 23.46	527.38 ± 39.88	0.559
Day 0 to 28	283.33 ± 16.49	310.42 ± 32.52	316.96 ± 38.80	315.92 ± 17.75	358.78 ± 26.49	0.453
ADFI (g/d)						
Day 0 to 14	373.39 ± 16.39a	448.60 ± 24.8b	436.21 ± 8.759b	435.14 ± 8.78b	488.54 ± 26.72b	0.006
Day 14 to 28	892.56 ± 38.65	937.97 ± 75.74	917.55 ± 79.88	973.08 ± 60.65	1092.76 ± 51.73	0.221
Day 0 to 28	632.98 ± 25.11a	693.28 ± 47.26ab	639.91 ± 19.86a	704.11 ± 34.41ab	790.65 ± 37.94b	0.029
F/G						
Day 0 to 14	2.33 ± 0.27	2.61 ± 0.24	2.87 ± 0.29	2.72 ± 0.21	2.63 ± 0.21	0.623
Day 14 to 28	2.13 ± 0.04	2.02 ± 0.02	1.90 ± 0.05	2.09 ± 0.06	2.10 ± 0.10	0.132
Day 0 to 28	2.25 ± 0.08	2.30 ± 0.14	2.25 ± 0.15	2.24 ± 0.06	2.24 ± 0.12	0.996

Data are presented as means ± SEM ($n = 6$). EO: essential oil; ADG: average daily gain; ADFI: average daily feed intake; F/G: feed/gain ratio. Different letters (a, b) indicated significant differences ($P < 0.05$).

Table S2. Primer pairs used for the real-time quantitative polymerase chain reaction.

Gene	GeneBank no.	Sequence (5'–3')	Product length, bp	Reference
β-actin	XM_003124280.5	F: CTGCGGCATCCACGAAACT	147	(64)

		R: AGGGCCGTGATCTCCTTCTG		
MUC1	XM_021089728.1	F: GGTACCCGGCTGGGGCATTG<br>R: GGTAGGCATCCCGGGTCGGA	146	(65)
MUC2	XM_021082584.1	F: CTGCTCCGGGTCCTGTGGGA<br>R: CCCGCTGGCTGGTGCGATAC	101	(65)
MUC4	XM_021068274.1	F: CATGGAGACCACCAGAGAAAC<br>R: GGTGTCCACTTGTGGAAGATAA	83	
ZO-1	XM_021098827.1	F: CCAACCATGTCTTGAAGCAGC<br>R: TGCAGGAGTGTGGTCTTCAC	215	(66)
claudin-1	NM_001244539.1	F: AAGGACAAAACCGTGTGGGA<br>R: CTCTCCCCACATTCGAGATGATT	247	(67)
occludin-1	NM_001163647.2	F: CAGGTGCACCCTCCAGATTG<br>R: GGACTTTCAAGAGGCCTGGAT	110	(68)

MUC1: mucin-1; *MUC2*: mucin-2; *MUC4*: mucin-4; *ZO-1*: zonula occludens-1.

Table S3. Effect of EO on piglet hematology index

	Control	0.04% B	*P*-value
WBC (10^9/L)	20.37 ± 1.93	19.14 ± 1.11	0.594
Neu (10^9/L)	5.29 ± 0.78	6.75 ± 1.78	0.937
Lym (10^9/L)	13.77 ± 1.44	11.31 ± 1.03	0.195
Mon (10^9/L)	1.10 ± 0.19	0.84 ± 0.11	0.280
Eos (10^9/L)	0.18 ± 0.05	0.22 ± 0.04	0.503
Bas (10^9/L)	0.02 ± 0.00	0.02 ± 0.00	0.713
Neu (%)	26.08 ± 2.88	33.67 ± 6.80	0.589
Lym (%)	67.63 ± 2.35	60.58 ± 6.56	0.699
Mon (%)	5.35 ± 0.64	4.48 ± 0.66	0.368
Eos (%)	0.80 ± 0.18	1.20 ± 0.29	0.268
Bas (%)	0.13 ± 0.05	0.07 ± 0.02	0.385

Data are presented as means ± SEM (n = 6). EO: essential oil; WBC: white blood cell count; Neu: neutrophil; Lym: lymphocyte; Mon: monocyte; Eos: eosinophil; Bas: basophil.
